# A novel APOC2 gene mutation identified in a Chinese patient with severe hypertriglyceridemia and recurrent pancreatitis

**DOI:** 10.1186/s12944-015-0171-6

**Published:** 2016-01-16

**Authors:** Jingjing Jiang, Yuhui Wang, Yan Ling, Abudurexiti Kayoumu, George Liu, Xin Gao

**Affiliations:** Department of Endocrinology and Metabolism, Zhongshan Hospital, Fudan University, Shanghai, China; Institute of Cardiovascular Science, Peking University and Key laborotory of Molecular Cardiovascular Science, Ministry of Education, Beijing, China

**Keywords:** Hypertriglyceridemia, APOC2 gene, Mutation

## Abstract

**Background:**

The severe forms of hypertriglyceridemia are usually caused by genetic defects. In this study, we described a Chinese female with severe hypertriglyceridemia caused by a novel homozygous mutation in the APOC2 gene.

**Methods:**

Lipid profiles of the pedigree were studied in detail. LPL and HL activity were also measured. The coding regions of 5 candidate genes (namely LPL, APOC2, APOA5, LMF1, and GPIHBP1) were sequenced using genomic DNA from peripheral leucocytes. The ApoE gene was also genotyped.

**Results:**

Serum triglyceride level was extremely high in the proband, compared with other family members. Plasma LPL activity was also significantly reduced in the proband. Serum ApoCII was very low in the proband as well as in the heterozygous mutation carriers. A novel mutation (c.86A > CC) was identified on exon 2 of the APOC2 gene, which converted the Asn codon at position 29 into Ala, followed by a termination codon (TGA).

**Conclusions:**

This study presented the first case of ApoCII deficiency in the Chinese population, with a novel mutation c.86A > CC in the APOC2 gene identified. Serum ApoCII protein might be a useful screening test for identifying mutation carriers.

## Background

The severe forms of hypertriglyceridemia or chylomicronemia are usually caused by genetic defects. However, in most patients, it remains a challenge to define the underlying cause. The clinical features of chylomicronemia include: recurrent episodes of pancreatitis, hepatosplenomegaly, lipemia retinalis, and eruptive xanthomata. So far, mutations in five genes have been found responsible for a portion of these patients, namely lipoprotein lipase (LPL), apolipoprotein A-V (APOA5), apolipoprotein C-II (APOC2), glycosylphosphatidylinositol-anchored high-density lipoprotein-binding protein 1 (GPIHBP1) and lipase maturation factor 1 (LMF1) [1]. Mutations in the LPL gene are the most common, while mutations in the APOC2 and LMF1 genes are rare.

The Apolipoprotein CII (ApoCII) protein circulates in the blood as a surface component of chylomicrons, VLDL, and HDL [2]. It plays an important role in triglyceride metabolism as a cofactor for LPL, the rate limiting enzyme for hydrolysis and removal of triglycerides from chylomicrons and VLDL. ApoCII deficiency leading to chylomicronemia was first described in 1978 and was recognized as an autosomal recessive disease [3, 4]. Patients with ApoCII deficiency have marked alterations of triglyceride metabolism, leading to elevated fasting triglycerides, chylomicrons, and VLDL [4].

To our knowledge, mutation of the APOC2 gene has never been described in the Chinese population. Here we describe a novel homozygous mutation of the APOC2 gene, leading to truncation and loss of the entire C-terminal, in a Chinese female patient with severe hypertriglyceridemia and recurrent episodes of pancreatitis. The patient had a favorable response to orlistat treatment.

## Methods

### Case description

A 31-year old female was referred to our hospital due to persistent hypertriglyceridemia. Her mother recalled that once she was ill at 2 years old and at a local hospital, her blood was “white”. Later she recovered and didn’t follow up. At the age of 19, she experienced first episode of acute pancreatitis after a feast. During that episode, serum triglyceride level was unknown. Two years later, she was attacked by pancreatitis again during pregnancy and her serum triglyceride level was above the 22.5 mmol/L upper limit at a local hospital. She recovered later and gave birth to a healthy baby boy. After delivery she was treated with fibrates, niacins and statins, to which she responded poorly.

On admission, no eruptive cutaneous xanthomas or lipemia retinalis were found. Routine laboratory tests, with the exception of plasma lipids (see results), were within the reference range. Abdominal Computed Tomography (CT) scan revealed slight hepatosplenomegaly. Despite high level of TG, her liver fat accumulation was relatively mild, quantified as 11 % by liver Magnetic Resonance Spectroscopy (MRS). Her physical and mental development appeared normal. Her parents were consanguineous (cousins). Her younger sister and brother, as well as her 10-year old son all appeared normal, without history of dyslipidemia or pancreatitis.

### Plasma lipid profile analysis

Blood samples were collected from the proband and her immediate family members after an overnight fast. Serum TG, T-C, HDL-C, LDL-C and non-HDL-C levels were measured enzymatically on an automatic analyzer (Hitachi High-Tech, 7600–120, Japan). The measurement of serum lipoproteins, including ApoAI(DiaSys, Germany), ApoB (DiaSys, Germany), ApoCII(Sekisui, Japan) and ApoE(Sekisui, Japan) was performed by immunoturbidimetric assays on an automatic analyzer (Hitachi High-Tech, 7600–120, Japan).

### Postheparin plasma LPL activity assay

Postheparin plasma (10 min after iv injection of 50 IU/kg heparin) was collected and total lipase activity was determined by incubation of the plasma at 37 °C for 60 min with 3H-triolein emulsion substrate prepared as described previously [5], with or without heat-inactivated rat serum as source of exogenous ApoCII. Lipase activity was mainly for hepatic lipase (HL) activity after LPL activity was inhibited with 1 M NaCl on ice for 30 min. LPL activity was calculated by subtracting HL activity from total lipase activity (1 mU corresponds to 1 nmol free fatty acid generated per minute).

### Analysis of candidate genes

Genomic DNA was extracted from peripheral blood leucocytes by a standard procedure. All exons, including the intron/exon boundaries of APOC2, LPL, LMF1, APOA5, and GPIHBP1 genes were PCR-amplified and sequenced as previously described [1]. Genotyping of the APOE gene was also performed by TaqMan assays following manufacturer’s instructions.

The study was approved by the ethics committee of Zhongshan hospital, Fudan University. Informed consents were obtained from each subject participating in the study after full explanation of the purpose and nature of all procedures used.

## Results

### Serum lipid and lipoproteins

**Fig. 1:**
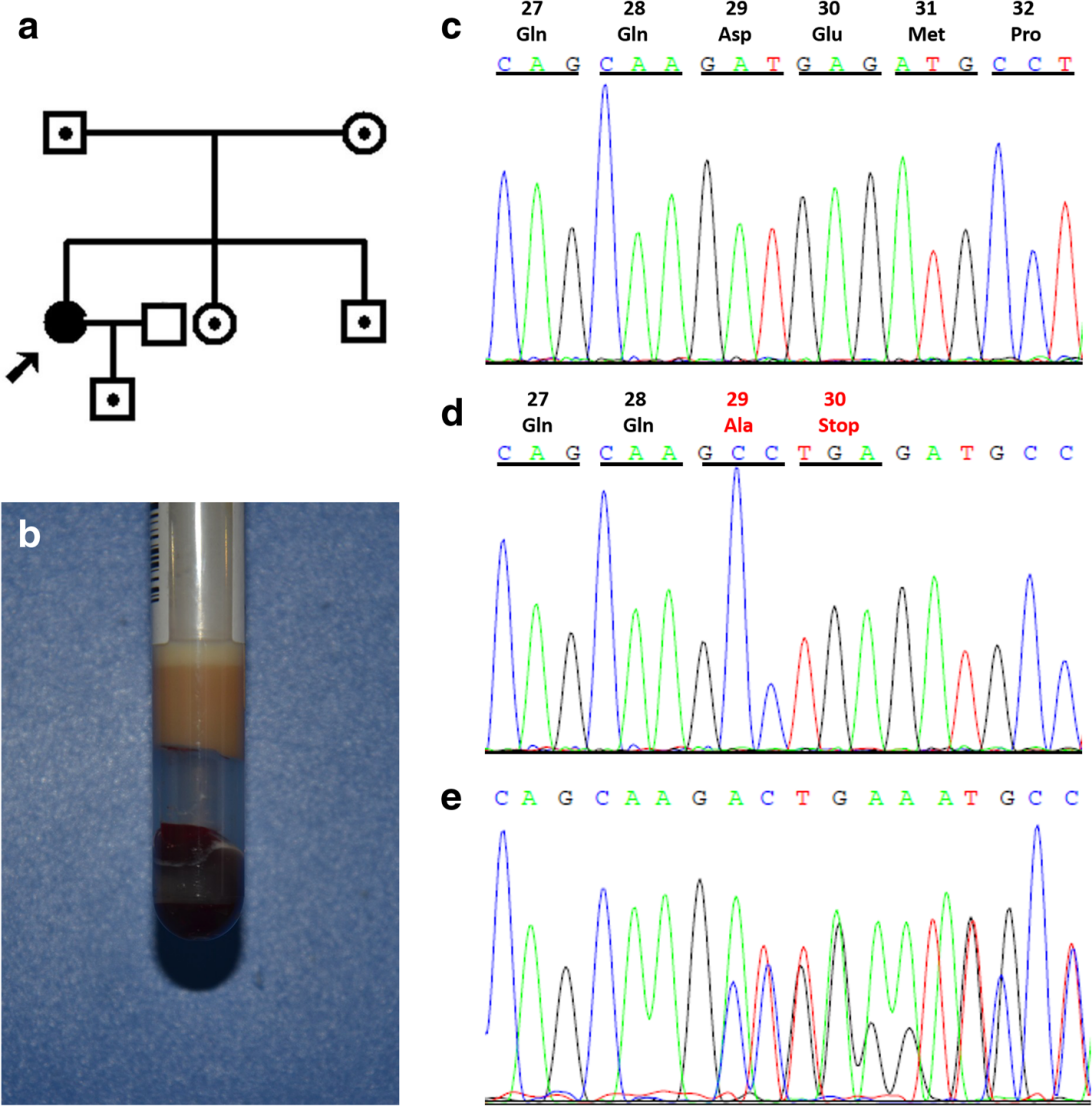
Pedigree and DNA sequence analysis of the family. **a** Pedigree of the family with APOC2 gene mutation. **b** Opacitas serum with a chylomicron layer on the top after placed overnight at 4 °C. **c**, **d**, **e** Electropherogram of a wild type control (**c**), the proband with homozygous APOC2 gene mutation (**d**) and her sister with heterozygous APOC2 gene mutation (**e**)

On admission, the proband’s serum TG, HDL-C and LDL-C were 17.42 mmol/L, 0.67 mmol/L and 0.36 mmol/L, respectively. The fasting serum after standing overnight at 4 °C had diffuse lipemia throughout and a chylomicron layer on top (Fig. 1b), typical of Fredrickson type 5 hyperlipidemia, suggesting high levels of both chylomicron and VLDL. Five days after strict diet control, serum TG, HDL-C and LDL-C were 12.41 mmol/L, 0.49 mmol/L and 0.48 mmol/L, respectively (Table 1). Though detectable, ApoCII was very low in the proband’s serum, whereas serum ApoE level was sharply increased, consistent with the increased level of serum triglyceride (Table 1).

**Table 1:** Lipid profile of the pedigree

	Father	Mother	Proband	Proband^a^	Sister	Brother	Son	Reference
Age	51	50	31	31	27	23	8	
TC(mmol/L)	5.05	4.12	3.74	2.81	4.11	3.72	4.69	<5.2
TG(mmol/L)	1.3	0.83	12.41	4.5	0.87	0.51	1.18	0.6–1.7
HDL-C(mmol/L)	1.35	1.56	0.49	0.53	1.82	1.28	2.03	>1.04
LDL-C(mmol/L)	3.11	2.18	0.48	0.91	1.9	2.21	2.12	<3.12
non-HDL-C(mmol/L)	3.7	2.56	3.25	2.28	2.29	2.44	2.66	2.08–4.14
ApoAI(g/L)	1.39	1.43	1.11	0.97	1.43	1.16	1.76	1.1–1.9
ApoB(mg/dl)	0.98	0.69	0.6	0.52	0.6	0.56	0.67	0.75–1.5
ApoCII(g/L)	0.4	0.4	0.35	<0.2	0.3	0.8	0.6	1.6–4.2
ApoE(mg/L)	37	30	76	53	32	24	33	29–53

^a^recent follow-up

All the immediate family members of the proband had normal lipid levels, including TG, TC, HDL-C, LDL-C and non-HDL-C. All family members had lower level of ApoB, except the proband’s father, whose LDL-C was close to the upper limit. Interestingly, though detectable, the ApoCII level was low in all family members (Table 1).

The proband was maintained on strict low-fat diet and later was advised to take orlistat 60 mg tid. Though she didn’t strictly follow the suggested regimen, her TG declined gradually below 10 mmol/L and later further improved within 5 mmol/L during the past one year. In a recent follow up, serum TG level was 4.5 mmol/L (Table 1).

### Postheparin plasma LPL activity

Plasma postheparin LPL activity of the proband (homozygous) was significantly lower than her mother (heterozygous) and the normal control (wild type). In contrast, the addition of exogenous ApoCII (rat serum) lead to a dramatic increase of the proband’s LPL activity (Table 2).

**Table 2:** Post heparin plasma lipase activity (mU/ml)

	Total	HL	LPL	Exogenous ApoCII
proband	32.2	20.2	12.0	without rat serum
mother	74.7	32.7	42.0	
control	64.7	23.3	41.4	
proband	75.3	18.1	57.2	with rat serum
mother	142.5	22.0	120.5	
control	155.8	23.0	132.8	

### Sequencing of candidate genes

A homozygous mutation on exon 2 of the APOC2 gene in the proband was identified: c.86A was replaced by 2 consecutive Cs(c.86A > CC), which converts the Asn codon at position 29 into Ala, followed by a termination codon (TGA) (Fig. 1d). The presence of this mutation was confirmed by sequencing at both directions. This mutation was not identified in 100 Chinese unrelated healthy controls, ruling out SNP. A heterozygous mutation was observed in the proband’s parents, her younger sister, brother and her son (Fig. 1e). Further subcloning and sequencing confirmed that they were all heterozygous mutation carriers. No mutation was identified on exons, as well as the intron/exon boundaries of LPL, LMF1, APOA5, and GPIHBP1 genes. The ApoE genotype of all family members, including the proband, was ε3/ε3.

## Discussion

ApoCII, together with ApoCI and ApoCIII, belongs to the ApoC family [2]. In humans, ApoCII is mainly produced by the liver. The APOC2 gene is located on chromosome 19 and encodes an 101-residue peptide [6]. Cleavage of the N-terminal 22-residue signal peptide yields the mature human ApoCII protein, which containing 3 amphipathic helices. The N-terminal is responsible for lipid-binding and the C-terminal helix is responsible for activating LPL [6]. So far only 14 deleterious APOC2 gene mutations have been described [7–20] (Table 3). All mutations resulted in loss of functional C-terminal, being unable to activate LPL.

**Table 3:** Summary of APOC2 gene mutations

Type	Nucleotide change	Amino acid change	APOC2 level	Ref.
promoter	c.-86A > G	N/A	undetectable	18
missense	c.1A > G	M1V(ApoCII Paris)	undetectable	11
missense	c.142 T > C	W48R(ApoCII Wakayama)	undetectable	14
missense	c.281 T > C	L94P(ApoCII Hongkong)	n.d.	15
nonsense	c.177C > G	Y59*(ApoCII Bari)	undetectable	9
nonsense	c.177C > A	Y59*(ApoCII Padova)	very low	12
nonsense	c.10C > T	R4*(ApoCII Paris2)	undetectable	17
nonsense	c.255C > A	Y85*(ApoCII Auckland)	undetectable	19
deletion	c.270delT	frameshift(ApoCII Toronto)	undetectable	8
deletion	c.118delG	frameshift(ApoCII Nijmegen)	undetectable	13
deletion	c.70delC	frameshift(ApoCII Jap/ven)	undetectable	20
gross-del	Loss of E2,3,4	untranslated(ApoCII Tuzla)	undetectable	16
insertion	c.274dupC	frameshift(ApoCII St.Michael)	n.d.	7
splicing	IVS2 ds G-C +1	c.55 + 1G > C(ApoCII Hamburg)	very low	10
del-ins	c.86A > CC	frameshift(ApoCII Shanghai)	very low	

The present mutation, which we designated ApoCII_Shanghai_, was predicted to translate into a truncated 29 amino acid peptide devoid of the C-terminal. The ApoCII level was very low in the proband’s serum possibly because either the protein itself was unstable and rapidly degraded, or the protein couldn’t be properly secreted. Similar ApoCII levels have been found in patients homozygous for other mutations (Table 3). Reports on the serum ApoCII protein levels of heterozygous relatives have been inconsistent. While ApoCII_Toronto_ [3], ApoCII_Wakayama_ [14], ApoCII_Auckland_ [19], ApoCII_hamburg_ [21] and the current ApoCII_Shanghai_ heterozygous relatives had lower serum ApoCII levels, ApoCII_Padova_ heterozygotes had ApoCII levels similar to unaffected family members [22]. Clinically, serum ApoCII levels are usually elevated in patients with hypertriglyceridemia. So low ApoCII combined with high TG levels might suggest ApoCII deficiency and the presence of low ApoCII levels in direct relatives even further increases this probability. In the present study, immunoturbidimetric assay was used to determine serum ApoCII levels. Although not as sensitive as radioimmunoassay, this method is less technically demanding and more applicable. It is also cost-effective and suitable for screening.

Paradoxically, both excess and deficiency of ApoCII are associated with hypertriglyceridemia. Transgenic mice overexpressing human ApoCII had marked hypertriglyceridemia and the ApoCII levels positively correlated with TG levels, due to impaired clearance and consequent accumulation of VLDL [23]. Recently APOC2 loss-of-function mutant mice were generated using zinc finger nucleases, which exhibited high TG levels as expected [24]. For patients with ApoCII deficiency, recurrent hyperlipidemic pancreatitis remains the major threat, while cardiovascular consequences are generally unremarkable. Due to uptake of chylomicrons by macrophages, hepatosplenomegaly is common, though fatty liver is actually mild, as in the present case.

Currently therapeutic interventions in ApoCII deficiency are still limited. Transfusion of normal plasma can provide temporarily sufficient ApoCII to activate LPL and normalize lipid profile [4]. Though not applicable as a routine therapy, this is still of clinical significance, because in contrast to LPL deficiency, ApoCII deficient patients could benefit from fresh plasma transfusion in case of acute hyperlipidemic pancreatitis. Another ideal cure is a biologically active synthetic ApoCII peptide. According to one report, the reduction in plasma triglycerides after a single injection persisted for 13–20 days [25]. Recently a novel bi-helical ApoCII mimetic peptide also showed promising effects in a mouse model [24]. Unfortunately, due to the rarity of the disease, commercialized synthetic peptides are not available in the clinic. Impaired clearance of chylomicron is the major cause of hypertriglyceridemia in ApoCII deficiency. Orlistat, an agent for obesity, inhibits gastric and pancreatic lipases in the lumen of the gastrointestinal tract to reduce the digestion and absorption of dietary fat, resulting in less intestinal chylomicron synthesis [26]. A small study including 5 patients with type V hyperlipidemia showed the addition of orlistat to conventional therapy resulted in a further 35 % reduction in triglycerides [27]. Interestingly, in the present case, orlistat markedly improved serum triglyceride compared with strict diet control alone. Though taken intermittently, orlistat brought the triglyceride level down to below 5 mmol/L and maintained well without rebound.

## Conclusion

In conclusion, we identified a novel mutation in the APOC2 gene. This was also the first case of APOC2 gene mutation diagnosed in the Chinese population. In patients with severe hypertriglyceridemia, serum ApoCII might be a good screening marker for identifying mutation carriers. Orlistat might be effective and worth trying in homozygous patients, though synthetic ApoCII peptides are to be expected in future.
