# Erratum to: A novel APOC2 gene mutation identified in a Chinese patient with severe hypertriglyceridemia and recurrent pancreatitis

**DOI:** 10.1186/s12944-016-0206-7

**Published:** 2016-02-29

**Authors:** Jingjing Jiang, Yuhui Wang, Yan Ling, Abudurexiti Kayoumu, George Liu, Xin Gao

**Affiliations:** Department of Endocrinology and Metabolism, Zhongshan Hospital, Fudan University, Shanghai, China; Institute of Cardiovascular Science, Peking University and Key laboratory of Molecular Cardiovascular Science, Ministry of Education, Beijing, China

## Erratum

After publication of the original article [1], the authors noticed two errors found in the abstract and in the ‘Results’ section. These errors have been corrected in the original article.

Firstly, “Exon 2” was incorrectly appearing as “Exon 3” in both the abstract and the ‘Results’ section. The authors previously didn't count exon 1 because it didn't encode any amino acids of the protein.

Secondly, the amino acid at position 29 was erroneously given as “Asn”, rather than the correct “Asp” in both the abstract and the ‘Results’ section.
